# Annotation of a hypothetical protein (WP_002969292.1) from Brucella abortus

**DOI:** 10.6026/97320630015315

**Published:** 2019-04-30

**Authors:** Kanchan Rauthan, Divya Goel, Sudhir Kumar

**Affiliations:** 1 Department of Biotechnology, Hemvati Nandan Garhwal University, Srinagar Garhwal, Uttarakhand-246174, India

**Keywords:** Annotation, hypothetical protein, Brucella abortus

## Abstract

Brucellosis is a zoonotic disease caused mainly by the bacteria belonging to the genus Brucella, most common of them is Brucella abortus. Genome sequencing of Brucella was completed in 2005. While majority of the proteins were assigned function, a large number of the peptides remained un-annotated and were referred as 'hypothetical'. These hypothetical proteins may contain crucial information about the biology and pathogenesis of the B. abortus. Therefore, it is of interest to annotate one such hypothetical protein as a multiple antibiotic resistance regulator protein, MarR. The physiological parameters, localization and the structural features were predicted for this protein which corroborated as the winged-helix type DNA-binding domain superfamily of transcription factors.

## Background

Brucellosis is a disease with many names such as malta fever, bang's disease, Gibraltar fever, undulant fever and many more. It has been a major cause of concern with almost 5,00,000 cases annually. Brucellosis is mostly found in domestic animals and it is caused by bacteria of Brucella species. Its transmission through untreated or contaminated milk is most common. Brucellosis is potential bioterror threat. Veterinary and abattoir accidents are another way of transmission. Brucella is a small gram-negative aerobic coccobacillus and a facultative intracellular parasite which includes B. abortus, B. melintensis, B. suis, B. neotomae, B. ovis, B. canis etc 1.

The genome of Brucella contains two chromosomes of 2.1 and 1.16 mbp, respectively. The sequenced B. abortus genome is reported to have 3,296 ORFs or genes. Out of these 3296 ORFs, 2,158 are situated on Chromosome I and 1,138 are on Chromosome II. It was also reported by the same group that B. abortus genome contains many short ORFs, which are less than 100 aa long 2. Brucella genome is unique because of the absence of plasmids and temperate phage. No evidence showing natural transfer of genetic material in Brucella has been recorded though very low occurrence of transduction has been shown 3. Comparative genomics study of B. abortus with B. melintensis and B. suis reveals that it shares more identity with B. melintensis than the latter 2. All the species of the Brucella genus share a conserved gene pool with diversity mainly because of recombinations 3.

A large number of protein sequences in Brucella genome have not been annotated and are deposited as hypothetical proteins. A search in the NCBI database using term 'hypothetical proteins' in Brucella abortus genome yielded about 581 protein sequences. We tried to annotate and assign function to most of them (Unpublished data). One of the sequences that got our interest was a hypothetical protein WP_002969292.1 from Brucella abortus and we decided to further investigate and annotate the sequence. A large number of in silico tools were employed to determine the structural and functional characteristics of the sequence. The hypothetical protein was identified as Multiple Antibiotic Resistance Regulatory (MarR) protein.

## Methodology

### Sequence retrieval and analysis:

Amino acid sequence of Hypothetical protein WP_002969292.1 from Brucella abortus (hereafter referred as BaHP) was retrieved from NCBI. Physiological parameters were calculated for this sequence by using protparam from ExPASy server 4. The putative localization of BaHP was predicted using CELLO 5 and CELL-P LOC 2.0 6. The protein sequence was subjected to protein blast using blastp program of NCBI 7 to search for similar sequences. Secondary structure analysis and fold recognition: Secondary structures of BaHP were predicted using SABLE 8 and J PRED server 9. PHYRE2 10 server were used for fold recognition analysis and InterProScan 11 was used to search architectural motifs of the BaHP.

### Structural modelling and analysis:

The structural modelling of BaHP was performed in a stepwise procedure from Swiss�Model against the proteins with similar sequence. The search identified crystal structure of Ruegeria pomeroyi (3CJN) MarR protein 12 as the template. The predicted 3- D model was downloaded and refined using MODRefiner 13. The refined structure model was chosen on the basis of the stereochemistry quality report generated using PROCHECK (used for inspection of ?/F Ramachandran plot) in PDBsum 14. It was also used to find out the residues responsible for dimerization of the BaHP molecule. The DNA binding residues were predicted by aligning the structure of BAHP with structure of RovA master virulence regulator (PDB id 4AIK) with promoter DNA 15.

### Phylogenetic analysis:

The full-length MarR protein sequences from different bacteria were obtained from NCBI and used to perform phylogenetic analysis using MegaX program 16. The tree was calculated using the Neighbour-Joining method.

## Results

### Sequence analysis of BaHP:

The search for protein sequences using word 'hypothetical' yield a large number protein sequences in the genome of B. abortus in NCBI which has not yet been annotated or characterized. One of the hypothetical proteins carrying accession number WP_002969292.1 showed similarity with MarR protein in protein Blast. The protein sequence of this hypothetical protein is:

MVKRASETDQRQSHVYLTQAGLQTIKAIEKSIRKTEKDMLKGLD

KKDRKSLLKMLSRMEGNLVLRGAARVADEPETEPQEDDEAE

### Physicochemical properties:

Protparam tool from Expasy server was used to analyse the hypothetical protein. It predicted the molecular weight of this protein at about 9685.04 Da and reported an unstable protein based on the instability index. The protein was show to be hydrophilic in nature with pI of 6.6 (Table 1) Localization server predicted this protein to be localized predominantly in cytoplasm (Table 2).

### Secondary structure analysis and fold recognition:

The secondary structures of the BaHP predicted using SABLE and J PRED server predicted 2 small β-sheets (E1 an E2) and 2 large a- helix (H1 and H2) with little variation in percentage of β-sheets to be at 7.1 % and that of a-helix at 49.4 %. Rest of the sequences were in the form of loops. First β-sheet E1 was predicted to be from Val 2 - Ala 4 (3 aa) and second β-sheet was from Gln 12 - Leu 17 (6 aa). The a-helix H1 ranged between Gln 19- Leu 40 (21 aa) and H2 ranged between Lys 45 - Val 63 (19 aa) (Figure 1a). JPRED tool predicted it to be a member of MarR family transcriptional regulator cluster. Phyre2 predicted the fold as DNA/RNA-binding 3-helical bundle that belong to the "Winged helix" DNA-binding domain superfamily. It also showed that that BaHP belong to the MarR-like transcriptional regulators family. The Phyre2 search predicted that BaHP share a good homology with crystal structure of transcriptional regulator, MarR family, from Silicibacter pomeroyi bearing PDB id 3CJN 12 which was confirmed by sequence alignment (Figure 1b).

Interproscan predicted the BaHP sequence to contain the Helix- Turn-Helix (HTH) motif and a HTH marR-type domain between amino acid no. 1 - 60 which belonged to a Winged Helix like DNA binding Domain superfamily. Interproscan result also showed the BaHP to have a DNA-template regulation of transcription biological function and predicted it to be a transcriptional regulator. Combination of these results implies that BaHP is a transcriptional regulator that belongs to the HTH-MarR type family of winged helix DNA binding protein superfamily.

### Structural modelling and analysis:

The structure model of BaHP was prepared with Swiss-Model using crystal structure of Ruegeria pomeroyi MarR (3CJN) as the template for modelling and refined using the MODrefiner. BaHP model contained 62 amino acids in each monomer (Figure 2a). The BaHP monomer consisted of two β-sheets (from aa 2-6 and 13-17) and two large a-helices (from aa 19-41 and 45-60). The 2 monomers are arranged as dimers with interface between helix 2 (Figure 2b). The alignment of the modelled BaHP structure with the template structure showed the conservation of the structural features even while they share only 32 % sequence identity (rmsd 0.063 for 111 aa) (Figure 2c). The modelled structure was validated using PROCHECK which put all residues in Ramachandran favoured region (Figure 2d). The residues involved in the dimerization as predicted by the PDBsum were plotted (Figure 2e). The detailed visualization of these residues in Pymol confirmed the interactions that result in dimerization of the molecule (Figure 2f).

### DNA binding residues:

The swiss model also predicted a similar structure for modelling of BaHP which is a virulence regulator RovA from Yersinia pestis 15. The crystal structure of RovA in association with a promoter fragment (4AIK) was used to identify the DNA binding sites in BaHP. Some of the residues of RovA protein which were involved in binding to the promoter were also present in BaHP suggesting that it could also bind to the promoter in similar fashion. Residue R11, Q12 and S13 of BaHP were in near vicinity of the promoter DNA and thus could probably bind to its own promoter (Figure 4).

### Multiple Sequence Alignment and Phylogenetic Analysis:

**Figure 3:**
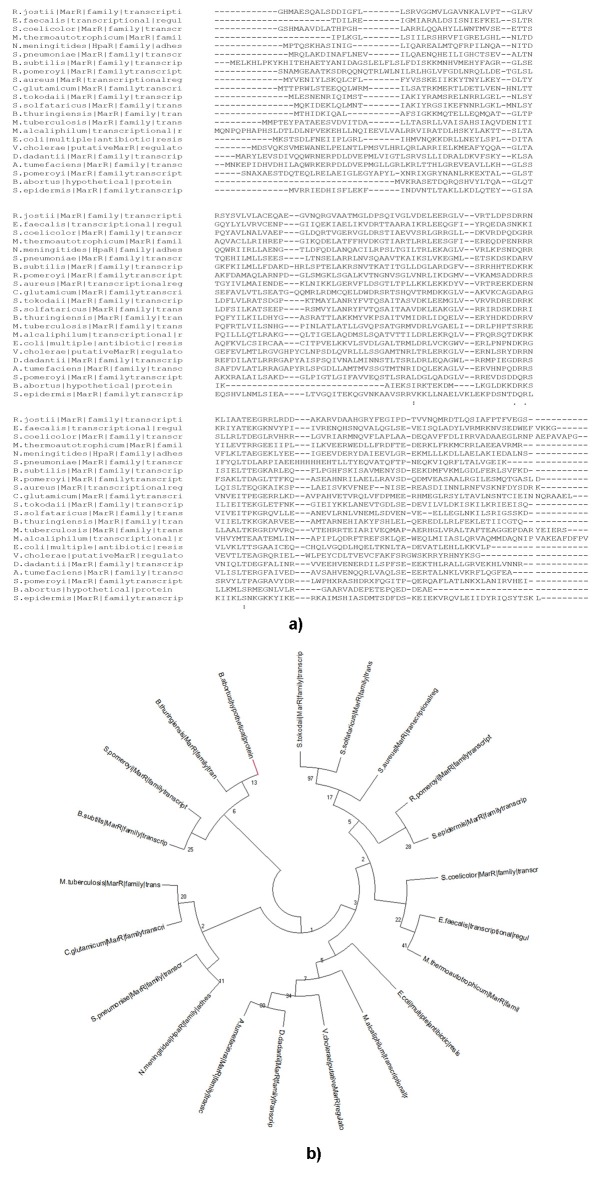
Sequence alignment and phylogenetic analysis. (a) The sequence alignment of MarR protein sequences form different organisms aligned to BaHP using Muscle Program (https://www.ebi.ac.uk/Tools/msa/muscle/). (b) The phylogenetic tree was inferred using the Maximum Parsimony method in MEGA X. The bootstrap consensus tree inferred from 300 replicates is taken to represent the evolutionary history of the taxa analyzed. Branches corresponding to partitions reproduced in less than 50% bootstrap replicates are collapsed. The MP tree was obtained using the Subtree-Pruning-Regrafting (SPR) algorithm with search level 1 in which the initial trees were obtained by the random addition of sequences (10 replicates). This analysis involved 21 amino acid sequences. There were a total of 190 positions in the final dataset.

**Figure 4:**
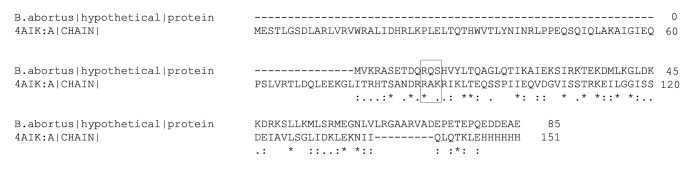
Alignment of BaHP and 4AIK protein sequences. A black Box highlights the amino acid residues in BaHP which are in place where the DNA binding residues of 4AIK are situated (virulence regulator RovA from Yersinia pestis) and might bind to the promoter DNA.

The sequence comparison of BaHP with different MarR sequences from different organisms using multiple sequence alignment shows non-significant sequence similarity. It is clear from the multiple alignments that the MarR superfamily is sequentially highly diversified and the BaHP also adheres to the same format (Figure 3a). Similar alignment with MarR proteins from Brucella abortus strain 9 also failed to show any sequence similarity with the hypothetical sequence, which implies that this protein is probably highly diversified. Phylogenetic and evolutionary relationships BaHP and other full-length MarR proteins were investigated using maximum parsimony bootstrap method. The BaHP here clustered with B. thuringiensis and B. subtillis MarR protein (Figure 3b).

## Discussion

MarR proteins, first identified in Escherichia coli, are the members of winged helix-turn-helix family of transcription factors 17 and they negatively regulate the operon that encodes for the drug efflux pump system. MarR binds to two direct repeats - sites I (predicted - 35 and - 10 promoter elements) and II (putative ribosome binding site) operator region (marO) separated by 21 bp in the marRAB promoter and inhibit the transcription of marRAB operon of E. coli. Mutation in MarR proteins result in the multiple antibiotic resistance phenotype 18. MarR exists as a homodimer protein, which generally assumes a triangular shape with pseudo-2-fold symmetry. The recognition helix of the wHTH domain binds the DNA major groove while the wing contacts the adjacent minor groove 17. The BaHP structural model shows a striking similarity to the typical MarR structures. The N- and C-terminal helices cross each other resulting in dimerization that also controls the DNA binding affinity of protein 19. The dimer can also be seen in BaHP structure and can potentially bind to promoter DNA.

## Conclusion

While the genome sequencing consortiums have lately resulted in influx of large genomic data, majority of protein sequences in these genomes remain un-annotated 20. These proteins, if investigated systematically can yield a better perspective into the general biology and pathology of the organism. We annotated one such hypothetical protein from Brucella abortus in this study. It was shown to be a member of MarR superfamily of promoter binding proteins using sequence and structural characteristics. The B. abortus MarR protein model revealed a highly conserved DNA binding domain (WHT domain) despite having low sequence similarity. Similar kind of approach can be used for annotation of other hypothetical proteins.

## Conflict of Interest

Authors declare no conflict of interest.

## Figures and Tables

**Table 1:** Physicochemical properties of hypothetical protein WP_002969292.1 calculated using Protparam.

NCBI Accession ID			WP_002969292.1
No. of Amino acids			85
Molecular weight, Mw (Da)			9685.04
Theoretical PI			6.6
Extinction coefficient			1490
Instability index	Computed	46.71
	Classification	Unstable
Aliphatic index			76.94
Grand Average Of Hydropathicity (GRAVY)			-0.978

**Table 2:** Prediction of Cellular Localization of the hypothetical protein WP_002969292.1 using different programs

Server	Sub cellular localization
CELLO	Cytoplasm
CELL-P LOC 2.0	Cytoplasm

**Figure 2:**
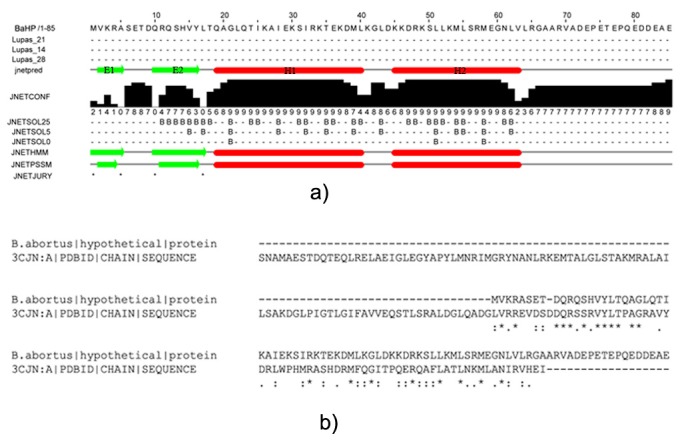
Sequence analysis BaHP for secondary structure prediction (a) The secondary structures as predicted by JPRED servers. (b) Sequence alignment of BaHP with FASTA sequence of 3CJN (crystal structure of transcriptional regulator, MarR family, from Silicibacter pomeroyi). The BaHP shared about 32 % sequence identity with the 3CJN sequence.

**Figure 2:**
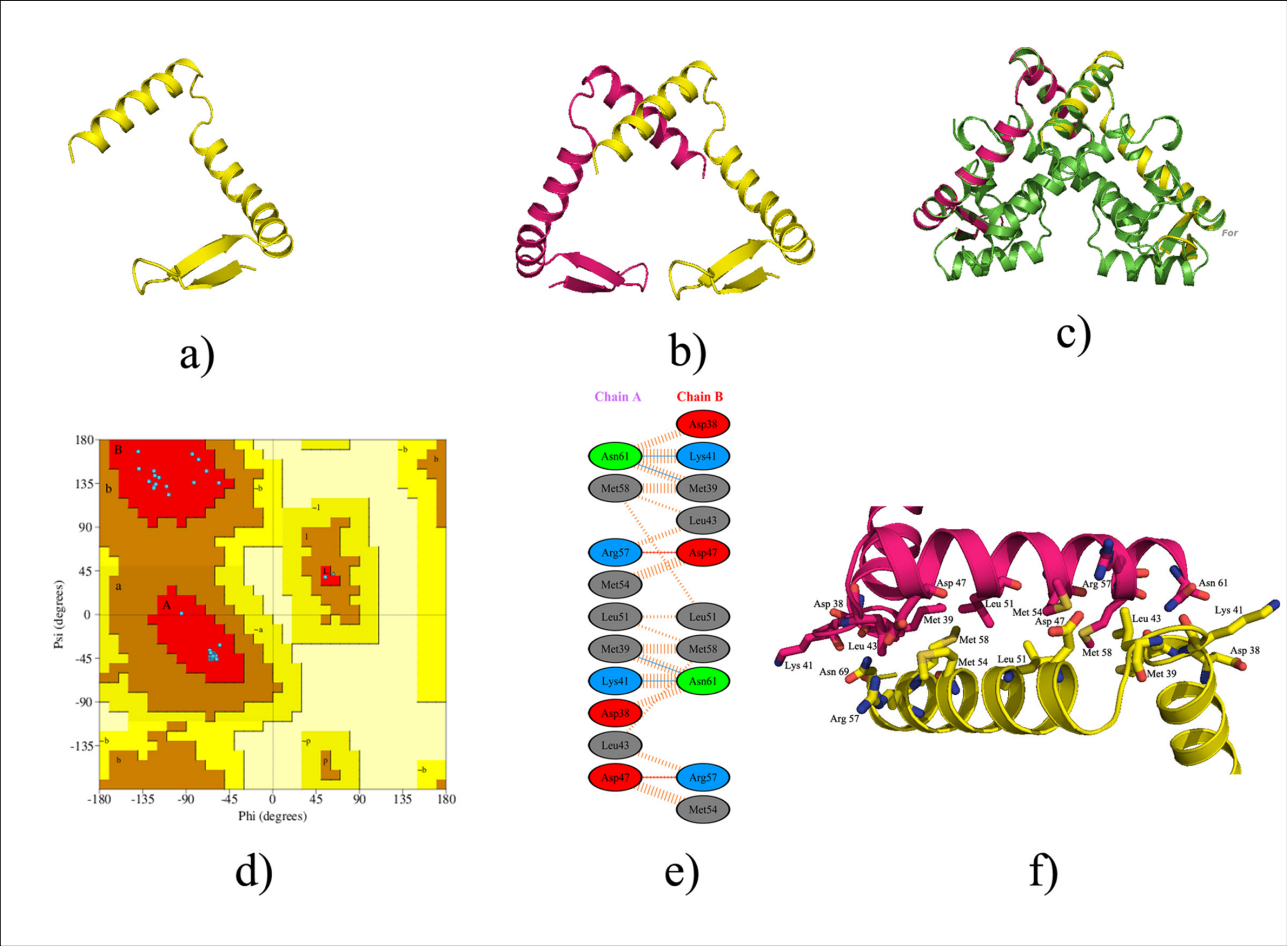
Structural Features of BaHP (a) Model of BaHP showing one monomer containing typical Helix turn Helix (HTH) domain; (b) Dimer of the BaHP model showing interactions between the C-terminal Helix 2 of both monomers; (c) Structural Alignment of BaHP model with its template 3CJN (Green); (d) Ramachandran plot for the predicted model showing 100% residues in allowed region; 9e) interacting residues at the dimer interface as predicted by PDBsum and (f) zoomed image to show the interactions of different residues in dimer interface
